# Molecular Probe Dynamics Reveals Suppression of Ice-Like Regions in Strongly Confined Supercooled Water

**DOI:** 10.1371/journal.pone.0044382

**Published:** 2012-09-26

**Authors:** Debamalya Banerjee, Shrivalli N. Bhat, Subray V. Bhat, Dino Leporini

**Affiliations:** 1 Department of Physics, Indian Institute of Science, Bangalore, India; 2 Dipartimento di Fisica “Enrico Fermi,” Università di Pisa, Pisa, Italy; 3 Instituto per i Processi Chimico-Fisici–Consiglio Nazionale delle Ricerche, UoS Pisa, Pisa, Italy; Bioinformatics Institute, Singapore

## Abstract

The structure of the hydrogen bond network is a key element for understanding water's thermodynamic and kinetic anomalies. While ambient water is strongly believed to be a uniform, continuous hydrogen-bonded liquid, there is growing consensus that supercooled water is better described in terms of distinct domains with either a low-density ice-like structure or a high-density disordered one. We evidenced two distinct rotational mobilities of probe molecules in interstitial supercooled water of polycrystalline ice [Banerjee D, et al. (2009) ESR evidence for 2 coexisting liquid phases in deeply supercooled bulk water. Proc Natl Acad Sci USA 106: 11448–11453]. Here we show that, by increasing the confinement of interstitial water, the mobility of probe molecules, surprisingly, increases. We argue that loose confinement allows the presence of ice-like regions in supercooled water, whereas a tighter confinement yields the suppression of this ordered fraction and leads to higher fluidity. Compelling evidence of the presence of ice-like regions is provided by the probe orientational entropy barrier which is set, through hydrogen bonding, by the configuration of the surrounding water molecules and yields a direct measure of the configurational entropy of the same. We find that, under loose confinement of supercooled water, the entropy barrier surmounted by the slower probe fraction exceeds that of equilibrium water by the melting entropy of ice, whereas no increase of the barrier is observed under stronger confinement. The lower limit of metastability of supercooled water is discussed.

## Introduction

Several water anomalies with deep implications in biology, atmospheric phenomena, geology, and food technology are rooted in the supercooled liquid state [1]–[6]. While there is wide consensus, with some controversy [7], [8], that water near ambient conditions is a uniform, continuous liquid [9], our understanding of water in the supercooled state below the freezing point is still widely debated.

### Models of supercooled water: an overview

The different viewpoints on supercooled water can be partitioned into two broad classes: mixture/interstitial models and distorted hydrogen bond or “continuum” models [10]. Mixture models consider that liquid water is composed of a small number of distinct components where molecules are surrounded by immediate neighborhoods with distinguishable structures. Whiting was the first to consider in 1884 liquid water as a mixture of a solid ice-like component and a normal liquid [11]. Later, mixture models with sharp distinction between “intact” and “broken” hydrogen bond (HB) were reported [12], [13]. However, the difficulty in specifying a few distinct states of liquid water motivated the growth of the continuum models. In this framework, first developed by Bernal and Fowler in 1933 [14] and Pople in 1951 [15], the picture of water structure is considered as a continuous distribution of approximately tetrahedral environments, corresponding to different degrees of distortion of the hydrogen bond (HB) ranging from strong HB's such as those in ice to highly distorted or even broken HB's [10]. The tendency to aggregation of unstrained ice-like polyhedra was also noted [1], [16] with increasing correlation length of the structure fluctuations [17]. An important new step about the structural aspects of water was the experimental observation of the phase transition between two different forms of amorphous ice by Mishima and coworkers [18]. The two amorphous ice phases were incorporated in the picture of the metastable and stable water by the liquid-liquid critical point (LLCP) scenario where [19]: i) liquid water displays polymorphism, i.e. it exists in two different phases, a highly-disordered high-density liquid (HDL), entropically favored, and a low density liquid (LDL) with local ice-like tetrahedral order, energetically favored, ii) the first-order LDL-HDL phase transition line terminates at a liquid-liquid critical point in the supercooled region. The LLCP scenario may be seen as a modern development of the mixture models. The universality of liquid-liquid phase transitions was argued in terms of two competing orderings, i.e. density ordering and bond ordering [20] leading in water to the formation of a rather stable ice-like locally favored structure in a sea of disordered normal-liquid structures [21]. To date, in addition to LLCP, three other separate thermodynamic scenarios have been proposed, i.e. the stability limit scenario [22], the singularity-free scenario [23], [24] and the critical-point free scenario [4]. It has been recently shown that LLCP scenario and the three other ones, including models that can reproduce more than one scenario [21], [25], [26], can be accounted for by one general scheme which predicts a LLCP at positive pressure [27].

### Current evidence of ice-like regions in supercooled water

The above discussion pointed out that regions of ice-like supercooled water are expected by mixture models of water [19], [21], as well as by the consideration of the strain energy of isolated elementary structural unit of hexagonal ice in a locale of strained and broken HB's [1], [16].

In parallel with several numerical studies, e.g. [17], [19], [23], [25]–[31], support to an increase in tetrahedrality and the presence of two different structural motifs in supercooled water is provided by a number of experimental findings. This includes discontinuities in the melting curve of high-pressure ice [32], changes in the local structure of both ambient water under pressure [33] and supercooled water confined in nanopores [34] or protein crystals [35], vibrational properties of nanoconfined water [36], enhanced density fluctuations in supercooled [37] and, controversially [8], [9], ambient water [7], bimodal mobility of guest molecules in interstitial supercooled water of polycrystalline ice [38], density hysteresis of nanoconfined heavy water [39]. However, consensus is not complete, e.g. on the existence of only two forms of amorphous water [40], the polyamorphism of liquid water [41], or the differences between bulk and confined water [4]–[6], [42]–[44].

### Water confinement in polycrystalline ice

At ambient pressure the supercooled regime of water ranges between the glass transition temperature 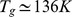 [3], [45], [46] and the melting temperature 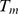. Above 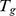 water transforms into a highly viscous fluid [3] crystallizing at 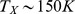. Since bulk water can be supercooled down to the homogeneous nucleation temperature 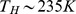, the region between 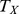 and 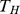 has been regarded as a region where liquid water is absent (“no man's land”, NML [2]). Nonetheless, the coexistence of crystals and deeply supercooled liquids was suspected already one century ago for bulk systems [47] (see also ref.[48]). More recently, the coexistence of ice and supercooled water was predicted by Nye and Frank [49], [50] and reported by experiments [51], [52], especially in the temperature range 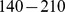 K [53]–[61], and by simulations in NML [29], [62]. Under suitable conditions the amount of liquid water in polycrystalline ice is not negligible. In the devitrification of vapor deposited solid only about 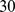
% of the material is transformed into cubic crystals of about 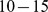 nm [55] and the remaining part has been identified as liquid [56]. Furthermore, simulations evidence still 15%–20% of liquid water between nanometer-sized ice crystals in NML [62].

In polycrystalline ice liquid water is localized where three grain meet in channels, or veins, that generally extend along the whole length of the grain edge. Four veins meet in a node (pocket) at a four-grain intersection, thereby forming a sponge-like, interconnected network of veins known as the vein system. The network was evidenced by experiments [51], [52], [61], [63] and simulations [29] and serve as interstitial reservoirs for impurities [51], [52], [58], [60], [61], [63], [65]–[69]. The vein width 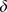 decreases with the temperature from the micrometer range very close to 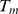 [51], [52], [63] down to dozens of nanometers at about 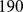 K [61].

Dimensional arguments lead to the conclusion that the volume fraction (f) of water with respect to ice in the vein system has the expression [59]:
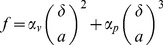
(1)

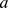
 is the average grain diameter, 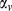 and 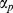 are dimensionless quantities depending on the geometry of the grain. The square term was derived by Frank [49], [70] who found 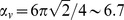 (the extra factor of four with respect to his result follows by considering both 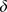 and 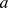 as diameters). The cubic term is the correction due to the finite volume of the pockets [59]. It was predicted [49], [50], [58], [59] and confirmed by experiments [51], [52] that the vein size 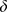 is determined by the thermodynamic properties like the relative surface energies of solid-solid and solid-liquid interfaces as well as by the temperature. Differently, the grain size is controlled by the thermal history. This aspect is discussed in detail in the next section.

### Thermal protocols for varying polycrystallinity

The experiments show that the size of the ice grains decreases by increasing the cooling rate [61], [64], [71]. The crystallization starting in the course of slow entrance into the supercooled region above 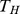 leads to macroscopic grains in the millimeter range [51]. Instead, much higher polycrystallinity is found in the ice formation following the devitrification of amorphous water above 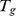 leading to small grains of about 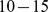 nm for thin films [55] or 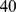 nm for thicker films [53]. This is in harmony with thermodynamic arguments leading to the conclusion that cubic ice particles with size of a few nanometers can coexist with water droplets of about the same size at temperatures in the 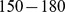 K range [60].

From the above discussion it follows that the liquid fraction in polycrystalline ice close to 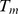 is quite small. Indeed, by taking 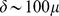
m and 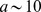 mm [51], one yields from Eq. 1 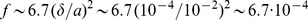, to be compared with the estimate 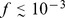 close to 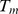 [49]. Distinctly, after quench cooling close to 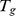 one has 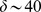 nm [61] and 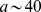 nm [53], [55]. This is consistent with the experimental finding 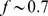 [56], and the anticipated coexistence of nanograins and nanodroplets of about the same size in the 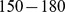 K range [60].

**Figure 1:**
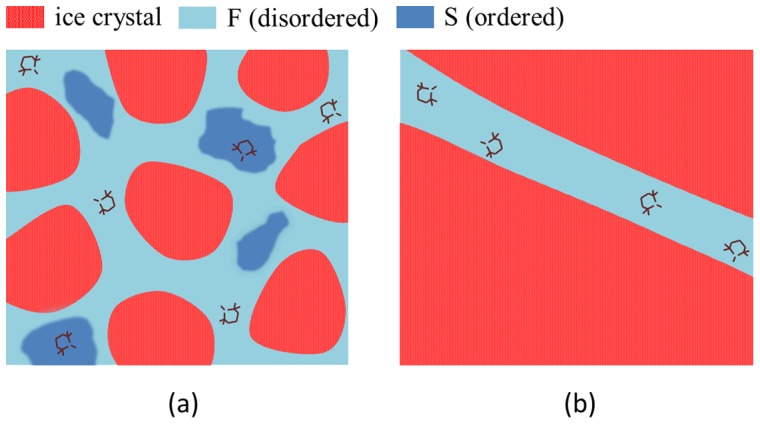
Two idealized ice/water mixtures with different polycrystallinity at 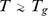. The scale of the pictures is the same. The two panels refer to the QRW (a) and the SC protocols (b), see text for details. The width of the liquid veins in the two mixtures is very similar and of the order of dozens of nanometers, whereas the size of the ice grains is 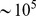 times larger in SC mixtures. Augmenting of the polycrystallinity increases the water fraction 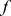 and reduces its confinement due to the additional paths and intersections. According to ref.[38] and the present study, ice-like patches (blue) with slow (S) mobility are included in the QRW liquid fraction. The patches are suppressed in the SC mixtures, leaving only the less ordered liquid fraction (light blue) with fast (F) mobility. The shape of the patches is unknown.

One may resort to the different character of the vein size (controlled by the thermodynamics) and the grain size (controlled by the thermal history) to control the degree of confinement of the liquid fraction in ice/water mixtures. Consider two ice-water mixtures with different polycrystallinity and equal temperature 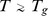, one resulting from the devitrification by rewarming previously quench-cooled water (quenched-rewarmed or QRW protocol), another with ice nucleated and grown close to 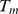 and then slowly cooled down to 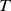 (slowly cooled or SC protocol), see Fig. 1. The mixtures have different grain size but very similar nanometric vein size. Thus, the devitrified sample with higher polycrystallinity exhibits a larger water fraction according to Eq. 1, which is less confined by the ice fraction due to the additional paths and intersections. Note that the exact temperature matching is not important to ensure very similar cross section of the vein 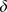 in QRW and SC ice/water mixtures. In fact, a near inverse-square dependence of 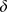 on the degree of supercooling is found [52]. This leads to a factor of 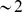 increase in 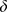 by rising the temperature from 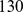 K to 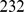 K.

It is worth noting that increasing the confinement of water close to a hydrophilic surface like ice is equivalent to a pressure (or density) increase [4], [6], [42]. This has the interesting consequence to unfavor the formation of unstrained, ice-like, hydrogen bond network in the confined water [42]. We also note that, according to recent simulations, strong confinement in hydrophobically nanoconfined water breaks cooperatively rearranging regions of 1 nm approximate size, facilitating the dynamics [72].

In a previous paper we reported evidence of two distinct rotational mobilities of probe molecules (spin probes) in interstitial supercooled water of polycrystalline ice [38]. The thermal protocol adopted in the sample preparation was the QRW protocol resulting, by devitrification, in a mixture of supercooled water and highly-polycristalline ice. It was speculated that the slow (S) and the fast (F) components of the probe molecules are trapped in the ice-like and the less ordered regions of the interstitial water, respectively (Fig. 1a). Here, we substantiate this claim by investigating the rotational mobility of the probe molecules in a water-ice mixture prepared by the SC protocol, i.e. by slowly cooling the sample from ambient conditions. The SC protocol yields ice with lower polycrystallinity than the QRW protocol and stronger water confinement (Fig. 1b).

The major conclusions of the study, which is presented and discussed below, are:

- the S fraction of the spin probes is embedded in regions of QRW water with ice-like structure (fig. 1a),
- the ice-like environment is suppressed in the liquid fraction of SC ice/water mixtures (fig. 1b).

## Results and Discussion

**Figure 2:**
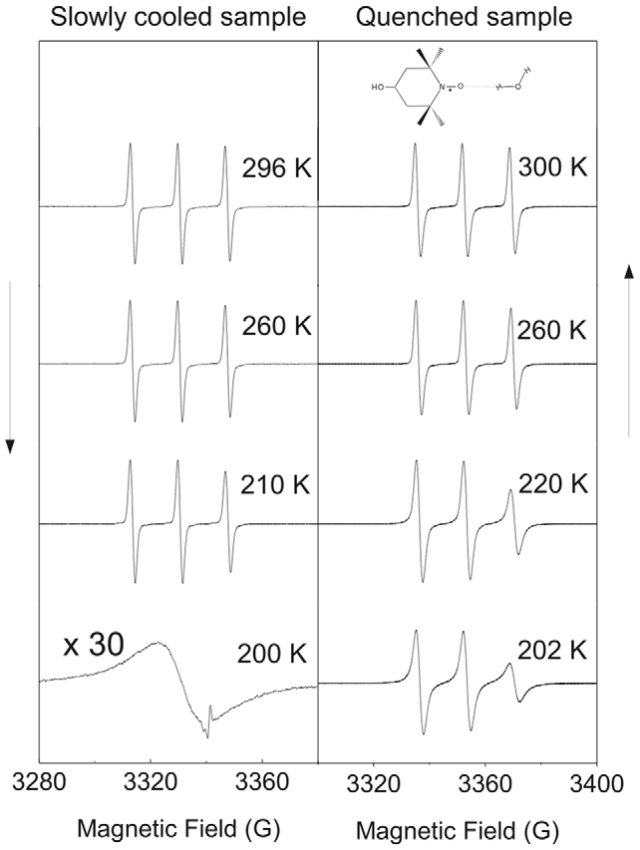
Structure and selected ESR lineshapes of the spin probe TEMPOL in water. Left: slowly cooled bulk water (SC protocol). Right: quenched and slowly re-heated bulk water at the indicated temperature (QRW protocol). The QRW sample contains ice with higher polycrystallinity. Note that the SC sample at 210 K exhibits narrower lines than QRW sample at 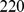 K, i.e. TEMPOL is rotating faster in SC water. The H-bonding of TEMPOL with water is shown in the top of the the right panel. Owing to the very weak ESR signal from TEMPOL in the SC sample at 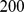 K, a small spurious signal from the quartz capillary used is observed at 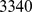 G.

We studied the rotational motion of the polar nitroxide molecule TEMPOL (spin probe) in the interstitial liquid water of polycrystalline ice by using the Electron Spin Resonance (ESR) spectroscopy [74], [75]. TEMPOL is a very stiff molecule and is coupled to water via hydrogen-bonds (HB), see Fig. 2 [73]. Due to the small size (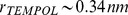 [38]), it is expected to perturb the water host in a limited way (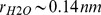). The sample preparation is described in Materials and Methods together with details about the ESR spectroscopy of spin probes, see also ref.[38], [75]. The guest molecule is expelled by the solid fraction and localizes in the interstices between the ice grains where the liquid water is trapped [38], [45], [58], [60], [66]–[69].

### Facilitated dynamics of TEMPOL in SC water

Fig. 2 presents the temperature dependence of the ESR signal of the spin probe in water prepared by the QRW and SC protocols, the former leading to ice with higher polycrystallinity. As usual, the lineshape, because of phase sensitive detection, is displayed in derivative mode by sweeping the magnetic field 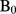 with constant microwave frequency 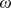 (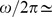 9.5 GHz in the present work).

Fig. 2 shows that, below about 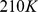 the ESR lineshape of TEMPOL in the liquid fraction of the SC sample changes abruptly and one observes a broad peak due to the strong exchange and dipolar interactions between very close TEMPOL molecules clustered in liquid pockets with mutual distances less than 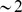 nm [38], [45], [66]–[69].

This finding is consistent with the stronger water confinement in the SC sample than in the QRW sample – where the lineshape collapse was never observed – combined with the shrinkage of the reservoirs where TEMPOL is trapped when departing from the melting point [52], [76].

Apart from the previous case, the ESR lineshapes in Fig. 2 are represented by three peaks. This pattern is characteristic of well isolated nitroxide probe molecules in a liquid host with no mutual interactions [38], [75]. The narrow width (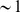 G) of each line of the triplet shown in Fig. 2 is due to the strong motional averaging of an otherwise broad (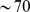 G) inhomogeneous ESR line (motional narrowing in liquid, for details see refs. [38], [75]). As a consequence, the *faster* the reorientation, the *narrower* the line. Inspection of Fig. 2 shows that TEMPOL rotates at comparable rates in SC and QRW liquid water at higher temperatures, whereas it becomes increasingly *faster* in SC water below 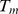 (compare the linewidths of the peaks, especially the rightmost one, of the ESR lineshape of TEMPOL in SC water at 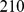 K with the corresponding ones of the QRW water at the *higher* temperature 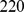 K). Due to the higher confinement of SC water with respect to QRW water, this finding is not trivial.

**Figure 3:**
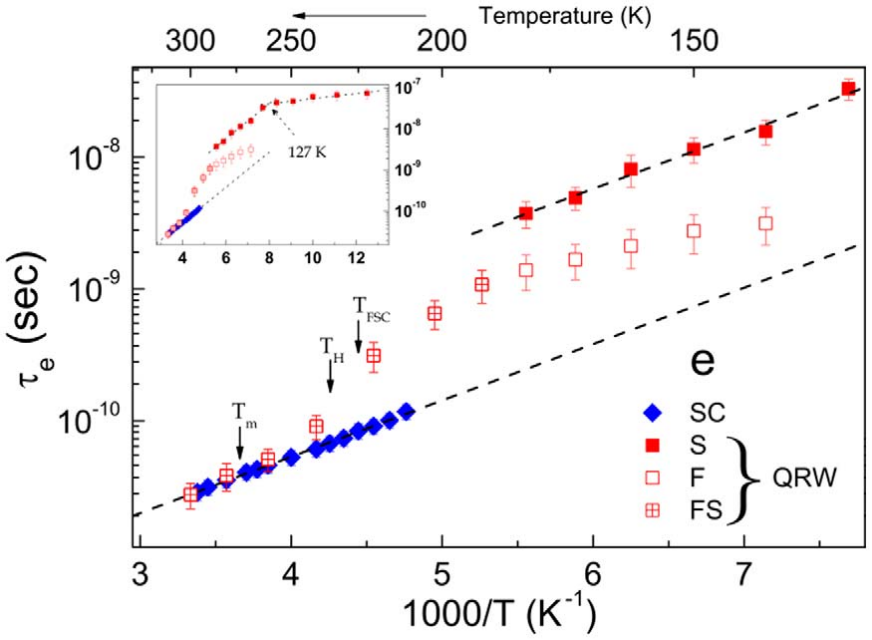
Rotational correlation time of TEMPOL in SC and QRW water. Part of the data are in the “no man's land” (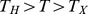). The two *parallel* dashed lines with slope 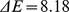 kJ/mol are the Arrhenius best-fit of the correlation times of TEMPOL in equilibrium water, SC water (blue) and in the low-mobility S fraction of the QRW water (red). The inset plots the data including the sub-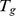 region. Note: i) the change of regime at 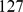 K close to 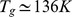, ii) the absence of any abrupt change at 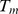 and, in SC water, at both 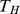 and 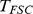.

To gain more quantitative insight, we fitted the ESR lineshape of TEMPOL by using the numerical methods detailed elsewhere [38]. Due to the globular shape of TEMPOL, only *one* adjustable parameter describes its reorientation in a given environment 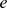: the rotational correlation time 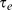, i.e. the area below the normalized time correlation function of the spherical harmonic 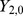. Roughly, 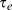 is a measure of the average time needed by TEMPOL to overturn. The temperature dependence of the rotational correlation time 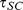 of TEMPOL in SC water is shown in Fig. 3 and compared to the one in QRW water, 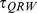 [38]. Before we go into the detailed comparison of 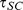 with the rich phenomenology of TEMPOL in QRW water, some preliminary remarks are in order. First, *no* signature of ice melting has been detected in QRW water while crossing 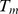, or of water freezing in SC water between 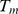 and 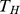. This is strong evidence that a negligible TEMPOL fraction is localized inside or close to the ice grains. Fig. 3 shows that the spin-probe reorientation in the supercooled region of QRW and SC water is strikingly different. The TEMPOL reorientation in SC water below 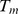:

- is *faster* than in QRW water (as hinted by Fig. 2),
- is driven by the *same* activated process as that of the equilibrium region (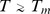),
- does *not* show the signature of the fragile-to-strong dynamic crossover (FSC) temperature at 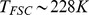 which is seen in QRW water [77].

Since TEMPOL links up with the HB network of water [73], the above findings point to facilitated dynamics of the SC water with respect to QRW water. By reminding that SC water is more confined by the ice grains than QRW water, support to this conclusion is provided by the finding that the formation of unstrained hydrogen bonds, limiting the fluidity, is inhibited in restricted environments [42].

### Energy and entropy barriers to TEMPOL reorientation

TEMPOL in QRW water between 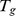 and 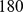 K is embedded in two environments where it exhibits fast (F) and slow (S) mobilities, see Fig. 3 and also ref.[38]. The situation is sketched in Fig. 1a. Above 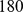 K the dynamical heterogeneity is averaged by the faster fluctuations and the ESR spectroscopy detects one *average* environment, denoted by FS (for simplicity labelled also as F in ref. [38]). It is intriguing to note that recent simulations of a monolayer of water adsorbed on a generic inert substrate evidence the disordering of the HB network above 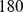 K [78].

TEMPOL, which rotates by breaking and reforming hydrogen bonds with water molecules after jumps of about 60° [38], [79], exhibits the same activation energy 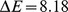 kJ/mol in the slow fraction of QRW water, the SC water and the equilibrium region (Fig. 3). This compares well with the activation energy to switch hydrogen-bond partners in pure (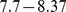 kJ/mol [80]) and doped (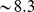 kJ/mol [81]) water. On this basis the TEMPOL reorientation is modeled as follows:

- TEMPOL switches water partner with an energy cost which is independent of both the water environment and the temperature.
- the additional temperature and environmental dependence of the reorientation rate is ascribed to the activation entropy 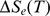 in the spirit of the transition-state theory.

We summarize the model by writing the TEMPOL reorientation time in a given environment 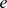 as:
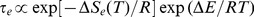
(2)with 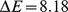 kJ/mol and 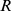 the ideal gas constant. Fig. 3 shows that, if TEMPOL is in SC water (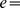
SC) or in the S fraction of the QRW water (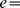
S), the activation entropy is temperature independent. This is not the case in either F (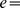
F) or FS (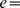
FS) environments of QRW water.

To understand how entropy limits the reorientation of TEMPOL in QRW supercooled water, we focus on the entropic barrier increase with respect to equilibrium, 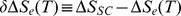, which is evaluated via Eq. 2 as:
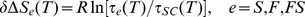
(3)


**Figure 4:**
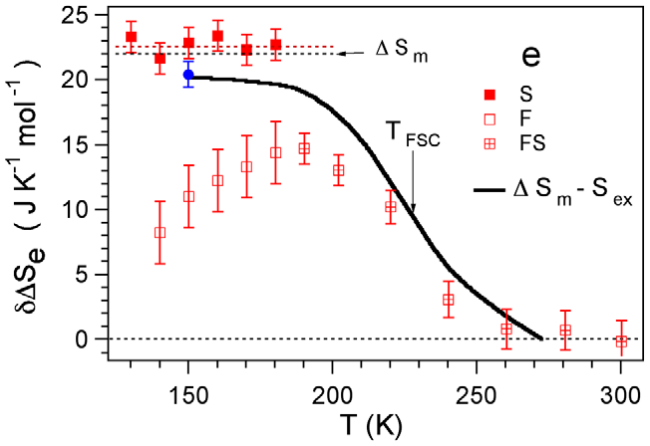
The entropy barrier to TEMPOL reorientation rising in supercooled QRW water. The dashed lines mark the entropy of melting 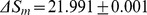 J K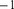 mol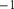 (black) and the best-fit value of the barrier of the slow fraction of TEMPOL 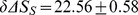 J K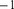 mol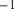 (red). The solid black line is a measure of the number of water configurations lost on cooling from 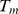 to 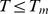, 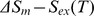, where 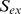 is the excess entropy of the liquid over the crystal [82]. The blue circle corresponds to 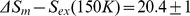 J K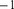 mol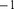 [84].



Eq. 3 assumes that the temperature dependence of 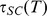 may be extrapolated below 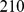 K. The results concerning 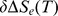 are shown in Fig. 4 and discussed below. Preliminarily, we define the quantity 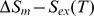 where 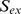 and 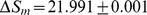 J K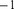 mol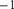 are the thermodynamic estimate of the excess entropy of the liquid water over the crystal [82] and the entropy of melting [83], respectively. We also resorted to the very recent measurement 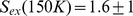 J K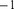 mol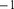 [84]. 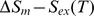 is a measure of the number of water configurations lost on cooling from 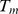 to 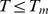 as it is seen by the relation [82]:

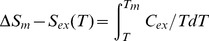
(4)where 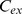 is the excess specific heat of the liquid water over the crystal.

### High-temperature entropy barrier

First, we discuss the temperature range 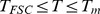. Fig. 4 shows that the increase of the activation entropy barrier 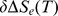 and the number of configurations lost by water, 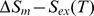, are very close to each other from equilibrium down to the fragile-to-strong crossover at 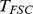 [86]. From this, it is evidence that the entropic barrier to be surmounted by TEMPOL to switch water partner in the OH bond is largely controlled by the spatial arrangement of surrounding water molecules, and the latter is negligibly perturbed by the probe molecule. It is interesting to compare this finding, referred to the guest-host coupling, to the sharp linear correlation between the entropy barrier increase from 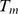 to 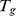, 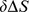, and the corresponding loss of the configurational entropy 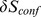 observed in glassforming systems [85]. More specifically, if a molecule – linked to the surroundings by 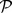 bonds – rearranges with the participation of 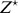 bonds, the approximate equality 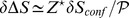 holds. In case of the hydrogen bonding between TEMPOL and water, by setting 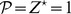 (see fig. 2) and by replacing 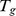 with a generic temperature 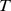 between 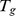 and 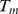, one recovers our approximate equality between 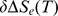 and 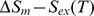.

### Low-temperature entropy barriers: ice-like regions in QRW water

We now discuss the temperature range 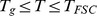. Below 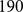 K ESR discriminates between the two TEMPOL fractions in the fast (F) and the slow (S) environments of QRW water, the latter with increasing weight on cooling [38] (the situation is sketched in Fig. 1a). From Eq. 3 (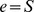) the entropic barrier of the S fraction is found to be constant, 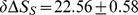 J K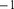 mol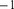 (Fig. 4). The fact that for 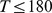 K, 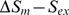 is lower than 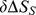 is ascribed (at least in part) to the positive contribution to 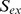 by less ordered environments.


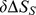
 agrees with the entropy of melting 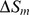 within 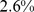 (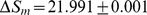 J K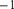 mol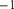 [83]), i.e. the activation entropy of TEMPOL in equilibrium water (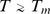) exceeds its counterpart in the S fraction of supercooled water by the melting entropy. In addition to the observed near coincidence between the activation entropy of TEMPOL and the configurational entropy of its surrounding water between 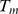 and 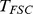, we take this finding as further evidence that water configurations control how TEMPOL switches from one hydrogen-bond water partner to another. More quantitatively, the present result is consistent with the conclusion that S liquid water maintains the fourfold coordination of ice within the ESR observation time 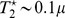
s [38]. Elsewhere, we argued that the S environment is not solid [38].

### Low-temperature entropy barriers: disordered regions in QRW water?

Below 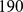 K, ESR reveals a fast (F) environment of QRW water (see Fig. 3 and a sketch of the situation in Fig. 1a). The entropic barrier of the F fraction of TEMPOL 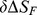 decreases by lowering the temperature (Fig. 4). The full characterization of the F fraction is made problematic by the fact that the weight of the ESR signal of TEMPOL in the F fraction decreases on cooling [38]. However, if we insist on assuming that 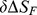 is a measure of the configurational entropy, one speculates that the F fraction is less ordered than the S one, i.e. has higher entropy than the S fraction (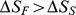).

### Lower limit of metastability of supercoooled water

Recent simulations of ice/water mixtures by Moore and Molinero evidenced the presence of threads and clusters of water molecules with local structure intermediate between ice I and liquid [29], [62]. This form of water, called intermediate ice [29], is thought to be a constitutive part of the structure of water at 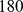 K [62]. At the beginning of the crystallization process the intermediate ice is already present and unrelated to crystal cores, whereas it localizes on the surface of ice crystallites at later stages. The major conclusion of ref. [29] is that the rate and mechanisms of ice formation is controlled by structural transformation leading to a sharp increase in the fraction of four-coordinated molecules in supercooled liquid water. An interesting consequence is that below 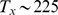 K (from classical nucleation theory) or 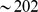 K (from numerical simulation) ice nuclei form faster than liquid water can equilibrate, i.e. water is not in a metastable state but is *out-of-equilibrium*. Said otherwise, 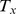 sets an effective lower limit of metastability of supercoooled water. Then, it is argued that in the range 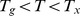 there is no metastable liquid water, but rather a less viscous liquid unable to relax before crystallizing [29].

Our experimental results, and their interpretation, put constraints to the above scenario. We reached temperatures lower than 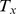 by the QRW protocol, i.e. quench-cooling to a state below 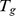 which is bound to be out-of equilibrium and then rewarming to the temperature of interest. Fig. 4 shows that the local structure of the S fraction of QRW water surrounding TEMPOL in the range 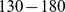 K is well equilibrated and close to ice. We remind that TEMPOL is *not* trapped in solid-state ice [38], [45], [58], [60], [66]–[69]. More experimental and numerical work is needed to clarify the matter. In particular we notice that the water model used in ref. [29] is a coarse-grained, monatomic model. It proved exceedingly useful and insightful in the investigation of several aspects of supercooled water's thermodynamics. However, the dynamics of this model is faster than in actual water because the barrier for breaking the hydrogen bonds is underestimated [62]. This barrier is involved in the mobility of water and the rate of crystallization. Then, the subtle interplay of crystallization dynamics and relaxation dynamics in the supercooled liquid could be not reproduced optimally in this model.

### Conclusion

In conclusion, we investigated the rotational dynamics of a probe molecule localized in the interstitial supercooled water of polycrystalline ice. The degree of confinement of the liquid water was found to vary according to the polycrystallinity of the ice. It is observed that the probe molecule has higher rotational mobility in water with stronger confinement. We interpret the probe dynamics in terms of a simple activated process with constant activation energy, due to probe's hydrogen bonding with water, and a suitable entropy barrier. We argue that the entropy barrier, which is due – through hydrogen bonding – to the configuration of the surrounding water molecules, yields a direct measure of the configurational entropy of the same. We find that, under loose confinement on supercooled water, the entropy barrier surmounted by the slower probe fraction exceeds that of equilibrium water by the melting entropy of ice, whereas no increase of the barrier is observed under stronger confinement. We conclude that loose confinement allows the presence of ice-like regions in supercooled water, whereas a tighter confinement yields the suppression of the water ordered fraction and leads to higher fluidity. Our results point to the striking conclusion that strengthening the confinement of water by ice destabilizes the hydrogen bond network of the liquid, even if one anticipates strong ordering induced by ice on water. These findings have broad implications on biology, atmospheric phenomena, geology, food technology as well as fundamental physics. In particular, they put constraints to recent numerical studies of the lower limit of metastability of supercoooled water.

## Materials and Methods

Samples were prepared in a capillary (dia 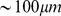) by doping a small amount of triple distilled water with about 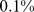 by weight of the polar radical TEMPOL (spin probe). TEMPOL accommodates well in water due to hydrogen-bonds and the moderate size (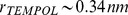 to be compared to 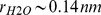).

The amorphous water samples (QRW protocol) were prepared by direct exposition to liquid helium (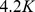) *in situ* in the ESR low temperature cryostat. The liquid helium transfer tube was modified such that a burst of liquid helium hits the capillary cooling it to 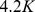 almost instantaneously leading to the formation of vitrified water.

The ESR signal of TEMPOL are recorded by using a X-band Bruker ER 200 CW EPR spectrometer. At a selected temperature no aging, i.e. no sample evolution, was ever detected.

The lineshape is evaluated by a stochastic memory-function approach [87], [88]. The reorientation of TEMPOL, due to its globular shape, is modeled by instantaneous random jumps with fixed size 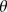 after a mean residence time 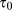 [89], as validated by theory [90] and simulations [91]. Under this hypothesis, the rotational correlation time 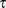 (the area below the normalized correlation function of the spherical harmonic 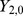 [75]) is given by 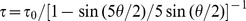. The temperature-independent magnetic parameters of TEMPOL were determined by the rigid-limit lineshape recorded at low temperature – where angular displacements are small [92]–[99] – according to a procedure detailed elsewhere [100].

The number of adjustable parameters of the theoretical lineshape changes over the temperature range under investigation. In general, the ESR lineshape of TEMPOL in QRW water is fitted by using two components, corresponding to the fast (F) and slow (S) fractions of TEMPOL, with weights 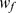 and 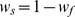, respectively. The S component depends on two adjustable parameters, i.e. 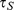 and 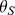, whereas, due to rapid motion, the F component depends on 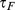 only. Therefore, to fit the ESR lineshape in the temperature region 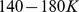 one needs four adjustable parameters (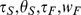). These reduce to two (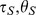) at lower temperatures where 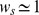 and one (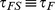) to higher temperatures where 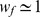. For TEMPOL in SC water only one fitting parameter (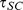) is needed. The theoretical lineshape was convoluted by a gaussian curve with width 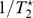 to account for the magnetic field produced by the rotating methyl groups close to the unpaired electron. 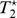 increases with the temperature from 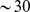 ns up to 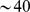 ns in the temperature range 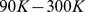.
